# One Pot Synthesis of Large Gold Nanoparticles with Triple Functional Ferrocene Ligands

**DOI:** 10.3390/ijms22052328

**Published:** 2021-02-26

**Authors:** Shenqing Wang, Fang Liu, Yin Liu, Hongyu Zhou, Bing Yan

**Affiliations:** 1 School of Chemistry and Chemical Engineering, Shandong University, Jinan 250100, China; 201520217@mail.sdu.edu.cn (S.W.); 201420234@mail.sdu.edu.cn (F.L.); yinliu@ucas.ac.cn (Y.L.); 2 Key Laboratory for Water Quality and Conservation of the Pearl River Delta, Institute of Environmental Research at Greater Bay, Ministry of Education, Guangzhou University, Guangzhou 510006, China; hyzhou001@gzhu.edu.cn

**Keywords:** stepwise synthesis, ferrocene derivatives, rapid quantification of ligands, low cytotoxicity GNPs

## Abstract

In biomedical, toxicological, and optoelectronic applications, the size of nanoparticles is one of the decisive factors. Therefore, synthesis of nanoparticles with controlled sizes is required. The current methods for synthesis of larger gold nanoparticles (GNPs, ~200 nm) are complex and tedious, producing nanoparticles with a lower yield and more irregular shapes. Using ferrocene as a primary reducing agent and stabilizer, sodium citrate as a dispersant, and sodium borohydride as an accessory reducing agent, GNPs of 200 nm were synthesized in a one pot reaction. Besides the roles of reducing agent and GNP stabilizer, ferrocene also served a role of quantitative marker for ligand loading, allowing an accurate determinate of surface ligands.

## 1. Introduction

The control of nanoparticle size in nanomaterial synthesis has been the focus of continuous efforts in the past decades. In particular, gold nanoparticles (GNPs) of various sizes were in high demand due to their size-dependent physicochemical and biological properties. They can be utilized in warious ways in photonics, catalysis, electronics, and biomedicine according to sizes. The most popular GNP synthesis method is the Turkevich-Frens method [1]. By this method, sodium citrate was used to reduce chloroauric acid to prepare aqueous solutions of GNPs of 12 to 70 nm as monodispersed spherical particles. Problems such as poor particle monodispersity and irregular shapes were often encountered when synthesizing larger GNPs with this method [2,3,4]. This greatly limits the exploration and utilization of large GNPs.

Since the suspensoid is a thermodynamically unstable system, a cause of failed synthesis is often the agglomeration of nanoparticles during the synthesis of larger nanoparticles. Current available methods include the selection of suitable reducing agents (such as citric acid, sodium citrate, ascorbic acid, hydroxylamine hydrochloride, sodium borohydride, and hydrazine hydrate) or stabilizers (for example, sodium citrate, glucose, and N-methylpyrrolidone) to improve stability of GNP suspension or to better control the particle size [5].

GNPs of 20 to 100 nm can be synthesized with a weak reducing agent sodium citrate or hydroxylamine hydrochloride. Ascorbic acid is also used as a reducing agent to synthesize GNPs with a size above 100 nm, but the highly toxic surfactant cetyltrimethylammonium bromide (CTAB) was used in the synthesis and they are hard to remove [6,7,8,9]. An issue with these methods is that spherical shape cannot be guaranteed. On the contrary, the products may present many different morphologies, such as irregular particles, oval or polyhedral nanoparticles [10].

A seeding-mediated synthesis strategy based on the temporal separation of nucleation and growth process is used to synthesize GNPs with diameters of 10 to 200 nm and can be obtained by the seed growth method [11]. However, to synthesize GNPs of 200 nm size, about 10 synthesis steps are needed [3], and each step requires cumbersome separation and purification operations.

Large GNPs have wide applications in biomedicine. For example, different endocytosis processes for larger GNPs (>200 nm) compared to smaller GNPs during air-blood-barrier crossing were discovered [12]. Polyethyleneimine (PEI 800 Da) modified GNPs with a size of 200 nm were applied as gene transfection vectors, showing high efficiency in gene delivery and transfection COS-7 cells with only slight cytotoxicity [13]. In view of so much promising applications, it is necessary to optimize the synthesis protocols of large GNPs.

To synthesize large GNPs with more sophisticated ligands, we here explored the possibility of using ferrocene derivatives as a reducing agent, a nanoparticle stabilizer, and a ligand quantitation marker simultaneously. We successfully achieved one pot synthesis of GNPs of 200 nm with ferrocene ligands, therefore, making significant progresses in the design and synthesis.

## 2. Results

### 2.1. Reaction Stage One: Ferrocene Reduced Chloroauric Acid to Form GNPs with a Diameter of 200 nm

**Figure 1:**
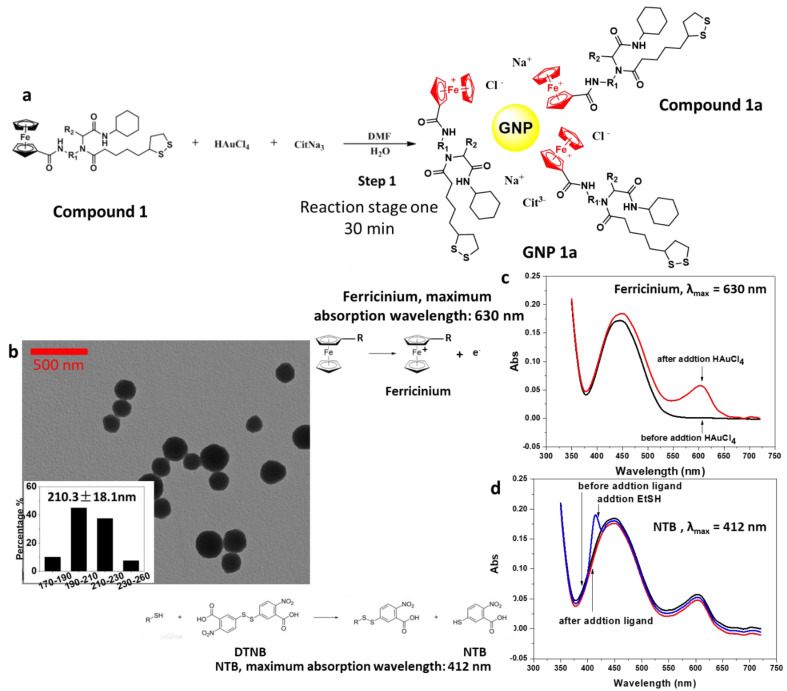
Reaction of ferrocene derivatives with HAuCl_4_ in stage one. (**a**) Reaction scheme. Au(III) obtains electrons and was reduced to Au(0) to form the initial gold nucleus. At the same time, the ferrocene structure in the ligand lost electrons and transformed into ferricinium (**Compound 1a**). (**b**) UV–Vis spectrum of the supernatant (λ_max_ = 630 nm) showed that ferricinium intermediates appeared during the reaction. (**c**) TEM images of the GNPs core showed GNPs core with diameters about 200 nm approximately (mid product). (**d**) UV–Vis spectrum of the supernatant (λ_max_ = 412 nm). No sulfhydryl groups appeared during the reaction of chloroauric acid with ferrocene.

The UV–Vis spectrum of the supernatant showed the absorption maximum of ferricinium at 630 nm (Figure 1c) after reduction. The supernatant of the reaction mixture was also tested by the Ellman reagent showing no sulfhydryl groups in solution (Figure 1d), indicating that –S-S- bond in compound bold was not broken until Au-S bond formed (Figure 1a).

TEM characterization of the reaction product showed that the GNP core had a diameter of 210.3 ± 18.1 nm (Figure 1b). The GNP products were electrostatically stabilized by ferricinium and accompanying citrate ions, which were added as stabilizes. Using citric acid instead of sodium citrate or using 1:1 ratio of these two reagents all produced irregular nanoparticles shapes or aggregates (Table S1). Here, citrate was not acting as a reducing agent because the oxidation of citrate to 1,3-acetonedicarboxylic acid needs to be carried out under high temperature (>363 K) in the presence of strong acid. The method involved in this paper was carried out at room temperature, and the reaction conditions were relatively mild. Therefore, citrate only served the role as a dispersant.

### 2.2. Reaction Stage Two: Ferricinium was Reduced to Ferrocene by Sodium Borohydride, and the Ligands Were Attached to the Surface of GNPs

**Figure 2:**
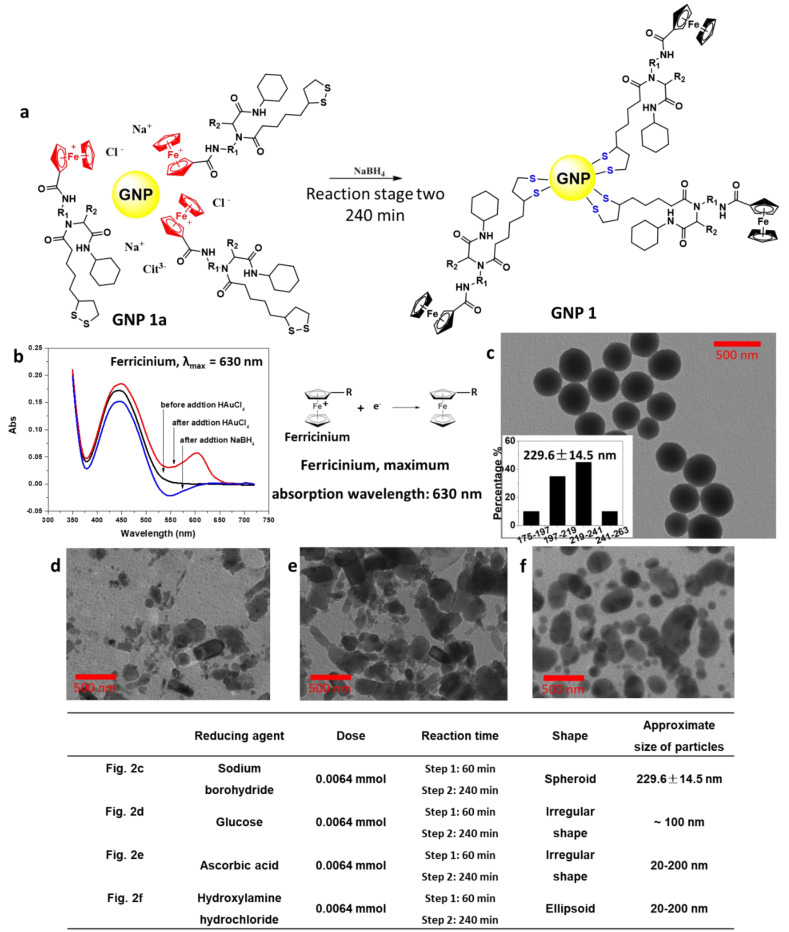
Reduction of ferricinium and formation of Au-S bond using NaBH_4_ in stage two. (**a**) Reaction scheme for stage two. (**b**) UV–Vis spectrum of supernatant (λ_max_ = 630 nm) showed that ferricinium intermediates appeared in 630 nm. (**c**) TEM images of the 200 nm GNPs. (**d**) Using glucose (0.0064 mmol) instead of NaBH_4_. (**e**) Using ascorbic acid (0.0064 mmol) instead of NaBH_4_. (**f**) Using hydroxylamine hydrochloride (0.0064 mmol) instead of NaBH_4_.

After addition of NaBH_4_, ferricinium was reduced to ferrocene in 240 min and the typical absorption peak of ferricinium at 630 nm disappeared (Figure 2b). At the same time, disulfide bonds in **Compound 1** were opened, reduced to thiols, and linked to GNPs via strong Au-S bond (Figure 2a). Finally, GNP with a diameter of 229.6 ± 14.5 nm was successfully synthesized (Figure 2c). Other reducing agents, such as glucose (Figure 2d), ascorbic acid (Figure 2e), and hydroxylamine hydrochloride (Figure 2f) were also tested in this reaction.

### 2.3. Universal Test of Ligand Structure

**Figure 3:**
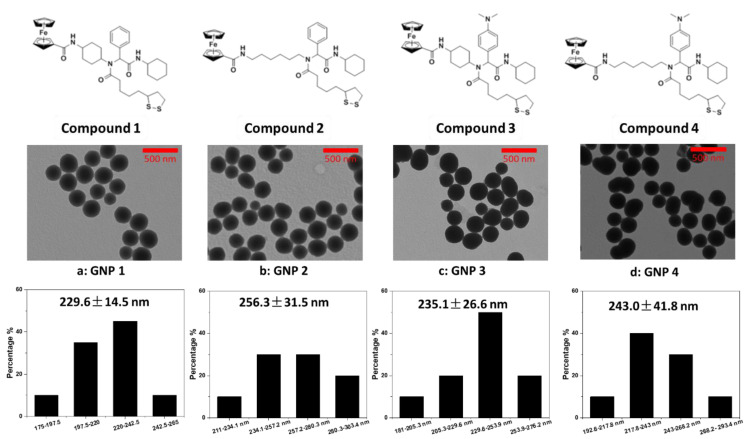
Structures of **Compounds 1–4** and TEM characterization of **GNPs 1–4**.

Considering the structure of small molecule organic ligands, it may affect the size and morphology of GNPs. Several ferrocene derivatives with different molecular structures were used in the test. The results (Figure 3 and Table 1) show that the change of molecular structure of organic ligands has no obvious negative effect on the normal reaction. Therefore, ligand **Compounds 1–4** were synthesized for **GNPs 1–4** preparation.

### 2.4. Using the Iron Atom in the Ligand Molecule to Complete the Rapid Determination of the Amount of Ligand Loaded on GNP

**Figure 4:**
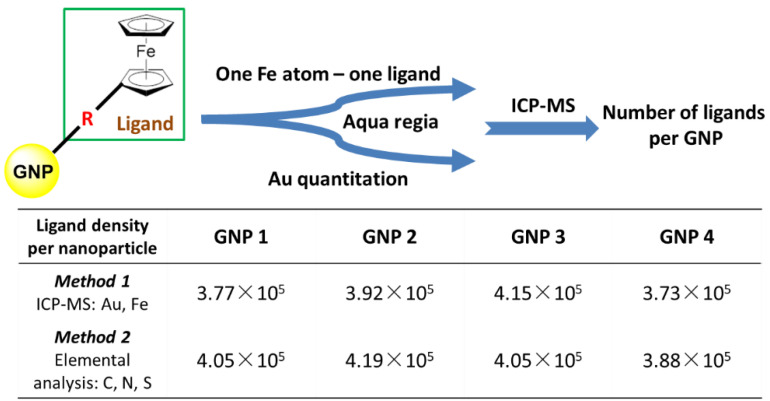
One-step determination of ligand loading on GNPs. The concentration of GNP was determined by the Au, and the concentration of ligand was determined by the Fe. Both determined using ICP-MS.

Based on the characteristics of ligand molecules, there is only one iron atom in the structure of each ligand molecule. It can be used to quantify ligands. After GNP was digested by aqua regia, it was determined by ICP-MS. The number of ligands was determined by the content of iron, and the number of gold nanoparticles was determined by dividing the total mass of gold (as determined by ICP-MS) by the mass of a single nanoparticle, as determined by its size. Thus, the rapid determination of ligand loading on GNP can be completed (Figure 4). We found that there were in average 3.8 × 10^5^ to 4.2 × 10^5^ ligands per GNP for **GNP 1**, **GNP 2**, **GNP 3** and **GNP 4**. All the aqueous dispersions of GNPs showed good dispersibility and stability for a long time (Figure S1).

### 2.5. Measurement of GNPs Cell Uptake

**Figure 5:**
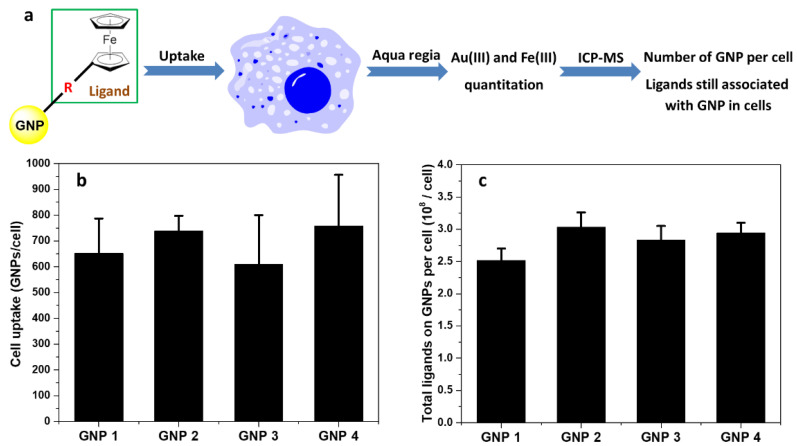
Cell uptake of GNPs. Cell uptake of A549 cells treated with GNPs. After treatment with GNPs (5 μg/mL) 24 h, cells were digested by aqua regia, and the content of Au(III) and Fe(III) was measured by ICP-MS. Error bars indicate mean ± standard deviation (n = 3). (**a**) The samples were digested by aqua regia and then tested by ICP-MS. (**b**) Based on the content of Au(III), the uptake of GNPs in cells was reflected. (**c**) Based on the content of Fe(III), the uptake of GNPs surface ligands in cells was reflected.

GNPs were used to treat the cells for 24 h. After counting the cells, they were completely digested by aqua regia, and then tested by ICP-MS (Figure 5a). The cell uptake of GNPs was confirmed by the contents of Au(III) (Figure 5b) and Fe(III) (Figure 5c). Based on these results, it can be concluded that the ligands loading on GNP surface did not dissociate in cells.

### 2.6. Cytotoxicity Test of GNPs.

**Figure 6:**
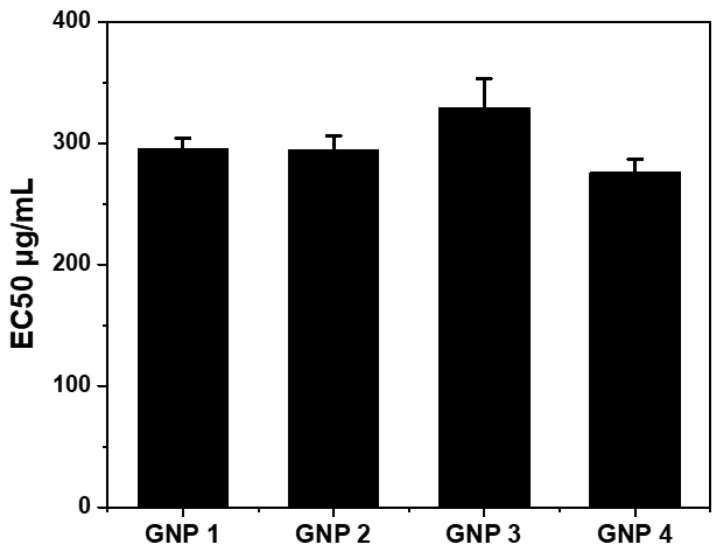
Cytotoxicity of GNPs. EC50 values of A549 cells treated with GNPs (based on Figure S2). After treatment with GNPs 48 h, cells were analyzed by CellTiter-Glo^®^ assay through luminescence. Error bars indicate mean ± standard deviation (n = 3).

GNPs were used to treat the cells for 48 h, and the cytotoxicity was tested by cell titer. All GNPs showed higher EC50 values (Figure 6) with considerable cell uptake. These four GNPs showed very low cytotoxicity and a reasonable dose-dependent relationship (Figure S2).

## 3. Discussions

Using conventional methods, such as using sodium citrate as the reducing agent, GNPs of 10 to 15 nm are first formed as “seeds” for larger GNPs synthesis [2,3,4]. Elemental gold continuously forms and the particle size gradually increases. Gold nanoparticles of 25 to 30, 40 to 45, 55 to 60, 70 to 80, 90 to 95, 110 to 115, 125 to 130, 150 to 160 and 180 to 200 nm can be obtained by multi-step reactions [3]. The larger the size of GNPs, the more reaction steps are needed. The corresponding purification and separation steps also becomes tedious. In this investigation, we explored the use of ferrocene compound as a novel reducing agent in a one-pot synthesis of GNPs with a diameter of 200 nm. Our synthesis method and reaction mechanisms involved are described below.

The homogeneous reaction between ferrocene derivatives and HAuCl_4_ at room temperature for 30 min reduced Au(III) to Au(0) with color change from pale yellow to purple (reaction I).

*n* AuCl_4_^−^ + 3*n* Fe(II) → 3*n* Fe(III) + 4*n* Cl^−^ + Au_*n*_ (reaction I).

The thermodynamics of the reaction can be estimated by the difference between the redox potential of AuCl_4_^−^ in aqueous solution AuCl_4_^−^ + 3e^−^ → Au (s) + 4Cl^−^ (*E*° = 0.93 V vs. SHE) and the redox potential of Fe(II) in ferrocene (*E*° = 0.545 V vs. SHE). By comparing the difference of electrode potential between the two, the reaction was feasible thermodynamically [14,15,16]. Fe(II) can be further oxidized by Au(III) to form Fe(III), stabilizing the newly formed GNP in solution.

Compared with sodium borohydride (*E*° = −1.24 V vs. SHE), the reduction activity of ferrocene was obviously weaker, and the reaction rate was also slower than that of sodium borohydride [17]. As a result, Au(III) will not be consumed rapidly and thoroughly as that when sodium borohydride was used. The Au(III) to Au(0) transformation and its continued deposition on the surface of the GNP “seeds” were progressing continuously and the particle size keeps increasing.

Dispersants play an important role in the preparation of GNP. The GNPs can be stabilized using citrate during the formation of GNP. At the same time, the aqueous solution of sodium citrate was alkaline, generating a suitable alkaline environment for the formation of GNP [18,19,20,21]. When sodium citrate was used as dispersant in proper concentration, the redox reaction between ferrocene group and chloroauric acid results in the formation of spherical GNPs with a diameter of 200 nm.

When the citrate dispersant was not used, the ferrocene group still underwent a redox reaction with chloroauric acid, but the resulting GNPs did not have spherical shape, suggesting the importance of citrate in maintaining spherical GNP shape [22].

In the reaction stage two, ferricinium was reduced to ferrocene by sodium borohydride. Other reducing agents, such as glucose, ascorbic acid, and hydroxylamine hydrochloride were also tested in this reaction. Based on the analysis of reaction effect, it suggested that their reducing activity was an important regulatory factor. The hydroxylamine hydrochloride has strong reducing ability (*E*° = −1.87 V vs. SHE). Its usage produced mixtures of both large particle (~200 nm) and small particle (~20 nm). The effects of glucose (*E*° = 0.05 V vs. SHE) and ascorbic acid (*E*° = −0.003 V vs. SHE) were not helpful, with products of irregular shaped gold nanoparticles.

As for why optimal result was obtained using ferrocene and only inferior results were obtained when using other reducing agents, we speculated that these might be due to different reducing mechanisms associated with different reducing abilities from various agents [23,24]. We believe that with further optimization, other reducing agents may achieve much better results. However, we did get very good results with ferrocene in combination with NaBH_4_ at current stage.

Physicochemical properties of nanomaterials have a significant impact on their biological effects. Surface charge, hydrophobicity, molecular geometry of ligands, hydrogen bonding, and π bonding by surface ligands are such factors [25]. It was desirable to explore these influencing factors through a combination of different substituents on surface ligands [26]. For example a comparison between the cyclohexane and the n-hexane structure reflects the influence of the molecular geometry. Comparison between the phenyl group and the N, N-dimethylphenyl group reflects the effect of π electron density and hydrogen bond receptors. These ligands also differ in hydrophobicity.

When ligand molecules were linked to GNP via Au-S bonding, they strongly affect the properties of GNPs [27]. Therefore, it was important to determine loadings of the ligand on each GNP. Here, we used ferrocene substituents as quantitative markers to determine the final loading of ligands on GNPs because one Fe atom corresponded to one loaded ligand. Accordingly, we analyzed digested solutions of GNPs for Au and Fe contents simultaneously and obtained a number of Fe atoms (ligands) per GNP.

The contact between nanomaterials and cells and the uptake of nanomaterials were the fundamental basis for all subsequent biological effects. At the same time, the stability of ligands loading on the surface of nanomaterials will also have a significant impact on the biological effects of nanomaterials. If the ligands loading on the surface of nanomaterials dissociate in cells, they may induce subsequent changes in biological effects.

The uptake of GNP based on gold and iron showed that the ligand loading on GNP did not dissociate in the intracellular environment (Figure 5). With the same mass concentration range, the cell viability was about 80% for all four GNPs, suggesting that there was no significant cytotoxicity and indicating that GNP may have potential of application in biomedicine and health care areas [5,28,29,30,31].

## 4. Materials and Methods

### 4.1. Synthesis of the GNPs

We have now investigated the ferrocene and HAuCl_4_ concentrations in view of conducting an effective reaction. Thus, an orange solution containing 0.0064 mmol ferrocene derivative (**Compounds 1–4**, Figure 4) in 20 mL DMF reacted with the 25 mg HAuCl_4_·3H_2_O and 1.9 mg sodium citrate in 2.25 mL water, at room temperature in 30 min without precipitation or agglomeration to provide a purple GNPs solution in DMF/H_2_O. Then, under the ice bath, 10 mg sodium borohydride in 15 mL water was slowly added to the reaction system through a peristaltic pump for 30 min. The solution color gradually became dark and red, and the reaction took 240 min to reach completion. Depending on the different ferrocene ligands, the GNPs were named **GNP 1**, **GNP 2**, **GNP 3** and **GNP 4**.

### 4.2. Zeta Potential and Dynamic Light Scattering (DLS) Measurements

GNPs were suspended in water with sonication. The DLS and zeta potentials of the GNPs were measured at 298 K using a Malvern Zetasizer instrument (Malvern Nano ZS90; Malvern Instruments Ltd., Worcestershire, UK). All samples were measured at the same concentration. Each sample was measured at least in triplicate.

### 4.3. Analysis of Ligands Loading on GNP

GNPs were suspended in water with sonication. Fifty μL GNP dispersions were mixed with 1 mL aqua regia. After completely dissolving, it was diluted with Milli-Q to 10 mL. The concentrations of Au(III) and Fe(III) were detected by ICP-MS. The concentration of GNP was determined by the Au(III), and the concentration of ligand was determined by the Fe(III). The ligands upload of GNPs was the ratio of the amount of ligands to the amount of nanoparticles (Figure 4).

### 4.4. TEM Images Characterization

TEM images of the GNPs were taken using a JEOL-1011 transmission electron microscope (JEOL, Tokyo, Japan) at 100 kV. The images were acquired using an AMT 2k CCD Camera.

### 4.5. Dispersion of Nanomaterials

The aqueous dispersion of the gold nanoparticles was sonicated and dispersed by sonication for 15 min. Sterile water was added to a concentration of 1 mg/mL to obtain a uniformly dispersed stock solution/mother liquor. After sterilization by batch sterilization, it was stored at 277 K for use. The ultrasound was evenly dispersed and diluted with the culture solution to prepare a desired concentration, which was vortexed and used for cell experiments.

### 4.6. Cell Culture

A549 is a human lung cancer cell line belonging to adherent cells. When the cells were cultured daily, RPMI 1640 culture medium containing 10% (*v*/*v*) fetal bovine serum was generally used, penicillin 100 U/mL was added, and streptomycin 100 μg/mL was added at the same time. It was placed in 310 K, 5% CO_2_ cell culture incubator. The cells were passaged at a concentration of 8.0 to 10.0 × 10^5^ per mL, and cells in the logarithmic growth phase were tested.

### 4.7. Cytotoxicity Test

For the experimental design, cells treated with RPMI 1640 complete culture medium were set as the negative control group, RPMI 1640 complete culture medium alone was used as the blank, and gold nanomaterial-treated cells were used as the experimental group. Dosing concentrations were 12.5, 25, 50, 100, 200 and 400 μg/mL, respectively. The concentration of the aqueous dispersion of GNPs used was 2 mg/mL. During the experiment, the mother liquor was sonicated for 3 to 5 min to achieve uniform dispersion. Afterwards, the gold nanomaterials were then diluted with RPMI 1640 complete medium to the dosing concentration. A549 cells in logarithmic growth phase were diluted to a concentration of 6 × 10^4^ mL with RPMI 1640 complete culture medium and inoculated in 96-well plates at 100 μL/well. After being incubated in a 310 K, 5% CO_2_ cell incubator for 24 h, the 96-well plate wells were aspirated, and 100 μL/well of GNPs with different concentrations was placed in the cell incubator. The cells were incubated for 48 h. RPMI 1640 complete medium, PBS, and CellTiter-Glo^®^ Assay reagents were equilibrated for 30 min to room temperature. The 96-well plate was removed from the incubator. The medium containing gold nanomaterials in each well was aspirated and equilibrated to room temperature PBS. It was then washed two times. In dark conditions, 50 μL of RPMI1640 Complete Medium and 50 μL of CellTiter-Glo^®^ Assay Working Fluid were added in sequence. The 96-well plate was placed on a shaker and gently shaken for 2 min. After standing for 10 min at room temperature in the dark, the mixture was aspirated at 70 μL/well, transferred to a white opaque 96-well plate for celltiter assay, and the fluorescence signal intensity was detected by the VICTORTM X2 Mulilabel Plate Reader. After reading the data, the background was deducted and cell survival was calculated. Each experiment was done in parallel three times.

## 5. Conclusions

In conclusion, we reported a new, rapid, efficient and simple method for the synthesis of ligand modified GNPs of 200 nm by using ferrocene derivatives and sodium borohydride as reducing agents at different reaction stages at room temperature. The method of rapid synthesis of GNPs and the flexibility of the GNPs design with respect to the novel ferrocene ligands make this one-pot homogenous reaction method very attractive. GNP with a size of about 200 nm has the opportunity to be used as a tumor targeting and drug carrier by providing an enhanced permeability and retention effect. Such large GNPs will also contribute to the investigation of size effect of nanoparticles in the structure-activity relationship elucidation. Furthermore, the ferrocene ligands with iron atoms provides quantification markers for surface ligands. The ferrocene ligands can also convert GNPs to regulatable redox nanomaterials.

## Figures and Tables

**Table 1:** Hydrodynamic diameter and zeta potential of **GNPs 1–4**.

	GNP 1	GNP 2	GNP 3	GNP 4
DLS/nm	266.2 ± 20.7	285.0 ± 9.2	251.8 ± 8.1	252.9 ± 13.6
Zeta potential/mV	−12.1 ± 0.3	−11.3 ± 0.5	−13.9 ± 0.2	−15.2 ± 0.6

## Data Availability

The data presented in this study are available in this paper.

## Supplementary Materials

The following are available online at https://www.mdpi.com/1422-0067/22/5/2328/s1.

## Abbreviations

CTAB	Cetyltrimethylammonium bromide
DLS	Dynamic Light Scattering
GNP	Gold nanoparticle
ICP-MS	Inductively coupled plasma massspectrometry
PBS	Phosphate Buffered Saline
SHE	Standard hydrogen electrode
TEM	Transmission Electron Microscope
