# Supplementary material for: One Pot Synthesis of Large Gold Nanoparticles with Triple Functional Ferrocene Ligands
Source: Int J Mol Sci. 2021 Feb 26;22(5):2328. doi: 10.3390/ijms22052328 (PMC7956544; doi:10.3390/ijms22052328)
Supplement: Supplementary file 1 [file ijms-22-02328-s001.pdf]

Supporting Information

One Pot Synthesis of Large Gold Nanoparticles with Triple Functional Ferrocene Ligands

Shenqing Wang, Fang Liu, Yin Liu, Hongyu Zhou, Bing Yan*

Contents

Figure S1. GNPs dispersed in aqueous solution and have good dispersion and stability.

Figure S2. Dose dependent effect of GNPs induced cytotoxicity.

Table S1. Effect of dispersant in stage one.

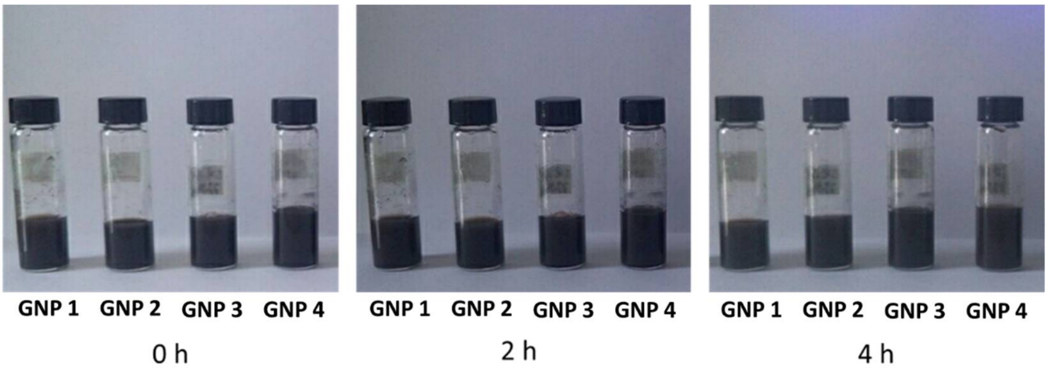

Figure S1. GNPs dispersed in aqueous solution and have good dispersion and stability. The aqueous dispersion of the GNPs was sonicated and dispersed by sonication for 15 minutes. The aqueous dispersion of GNPs is uniform and stable.

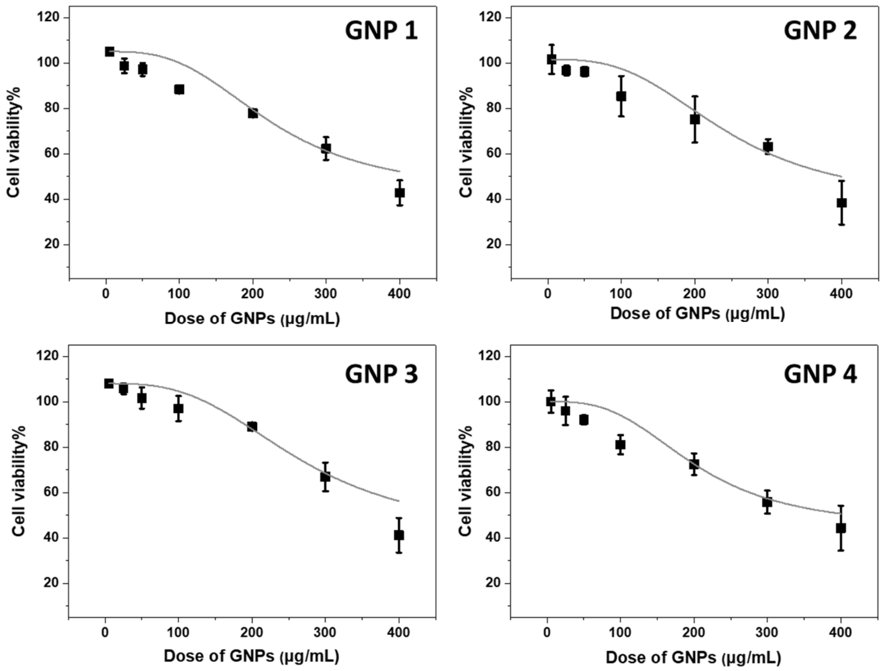

Figure S2. Dose dependent effect of GNPs induced cytotoxicity. Dosing concentrations were 12.5, 25, 50, 100, 200 and 400 μg/mL, respectively. After treatment with GNPs 48 h, cells were analyzed by CellTiter-Glo® assay through luminescence. Error bars indicate mean±standard deviation (n=3).

Table S1. Effect of dispersant in stage one.

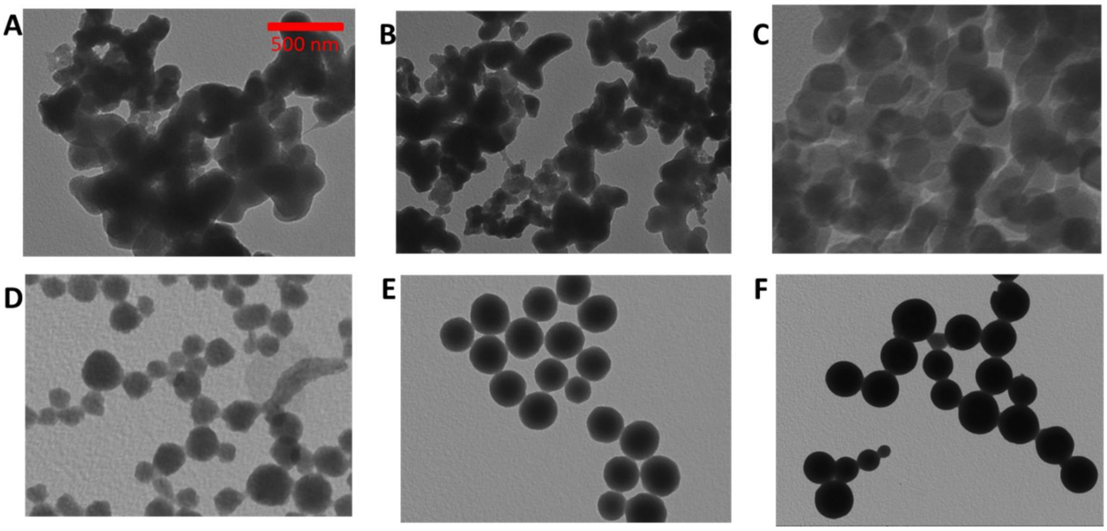

	Dispersant	Dose	Reaction time	Shape	Approximate size of particles
A	Null	0	Step 1: 60 min<br>Step 2: 240 min	Polygon	~ 100 nm
B	Citric acid	0.0064 mmol	Step 1: 60 min<br>Step 2: 240 min	Irregular shape	~ 200 nm
C	Sodium citrate<br>+ Citric acid	0.0032 mmol<br>+0.0032 mmol	Step 1: 60 min<br>Step 2: 240 min	Polygon	~ 200 nm
D	Sodium citrate	0.0032 mmol	Step 1: 60 min<br>Step 2: 240 min	Ellipsoid	~ 150 nm
E	Sodium citrate	0.0064 mmol	Step 1: 60 min<br>Step 2: 240 min	Spherical	~ 200 nm
F	Sodium citrate	0.0096 mmol	Step 1: 60 min<br>Step 2: 240 min	Spherical	~ 200 nm
